# A Qualitative Study on Organizational Factors Affecting Occupational Accidents

**Published:** 2017-03

**Authors:** Davood ESKANDARI, Mohammad Javad JAFARI, Yadollah MEHRABI, Mostafa Pouya KIAN, Hossein CHARKHAND, Mostafa MIRGHOTBI

**Affiliations:** 1. Dept. of Occupational Health, School of Public Health, Shahid Beheshti University of Medical Sciences, Tehran, Iran; 2. Dept. of Epidemiology, School of Public Health, Shahid Beheshti University of Medical Sciences, Tehran, Iran; 3. Dept. of HSE, Arya Sasol Petrochemical Company, Bushehr, Iran; 4. Dept. of Basic Sciences, Faculty of Nutrition Sciences and Food Technology, Shahid Beheshti University of Medical Sciences, Tehran, Iran

**Keywords:** Occupational accident, Organizational factors, Qualitative research

## Abstract

**Background:**

Technical, human, operational and organizational factors have been influencing the sequence of occupational accidents. Among them, organizational factors play a major role in causing occupational accidents. The aim of this research was to understand the Iranian safety experts’ experiences and perception of organizational factors.

**Methods:**

This qualitative study was conducted in 2015 by using the content analysis technique. Data were collected through semi-structured interviews with 17 safety experts working in Iranian universities and industries and analyzed with a conventional qualitative content analysis method using the MAXQDA software.

**Results:**

Eleven organizational factors’ sub-themes were identified: management commitment, management participation, employee involvement, communication, blame culture, education and training, job satisfaction, interpersonal relationship, supervision, continuous improvement, and reward system. The participants considered these factors as effective on occupational accidents.

**Conclusion:**

The mentioned 11 organizational factors are probably involved in occupational accidents in Iran. Naturally, improving organizational factors can increase the safety performance and reduce occupational accidents.

## Introduction

Today, people are facing many risks and difficulties when trying to earn money that is often associated with the development of science and technology. These risks increase occupational diseases and work-related accidents (1, 2). “An occupational accident is an occurrence in the course of work causing physical or mental occupational injury” (3). Occupational accidents are the reason of about 321000 fatalities and 317 million injuries worldwide each year. Work-related injuries are considered as a major health problem in Iran. Fatal and non-fatal accidents ratio are 0.95 in 100000 and 253 in 100000, respectively; since occupational accidents impose heavy costs on the economy of any country and it is important to protect the health of workers this large segment of society (4, 5). There are many factors associated with accidents. The analysis of large events such as Long Ford and Piper Alpha accidents indicated that technical, human, operational and organizational factors were influential on the sequence of accident (6, 7). Reporting Major Accidents Database showed that in 1998, in 64% of identified accidents in Europe, human factors (11%) and organizational factors (53%) contributed to accidents (8).

Manager and employees are two main components of an organization, which can play an important role in the safety of organization. Workplace accident is the result of employer negligence or employee carelessness (9). Employers have a crucial role in preventing workplace accidents; they should give importance to occupational health and safety, take preventative measures, and ensure that employees have the necessary information, training, and supervision to carry out their jobs safely. Employees should be conscious, make sure about the accidents, and perform their obligations regarding work safety while working (10). Therefore, to achieve higher safety in an organization, management and employees must perform their safety-related tasks.

There is a relationship between organizational factors and the employees’ safety behavior (11–13). Relationship between organizational factors and safety performance was studied on Taiwanese and Japanese oil refinery workers. The results indicated different organizational factors of the two countries as effective on the employees’ safety performance (14). The effects of organizational factors on safety self-efficacy, safety awareness, and safety behavior was studied in Jordanian companies, and showed that management commitment significantly affects safety self-efficacy and safety awareness (15).

Human and organizational factors were compared as predictors of frequency of accidents. Both human and organizational factors helped the frequency of the occupational accidents, also had a stronger effect on accident frequency (16). The relationship between individual, psychological, organizational and work-related environment variables on occupational accidents was examined by using structural equation modeling. Each variable has effect on their occupational accidents. Individual variables, including the healthy behavior and health mediated indirect effects of organizational variables (12).

Naturally, there are other organizational factors affecting occupational accidents identified. A research into this matter using a qualitative technique that includes multiple methods of data collecting and emphasizes reality experience can help us to obtain significant and comprehensive data; it will also make clear the existing background and the actual situation surrounding the formation of occupational accidents. Thus, this qualitative study was conducted based on the experience of experts, who form the core of provision.

## Materials and Methods

The present study attempt to provide a list of organizational factors related to the accident in order to facilitate conducting further studies in the field of controlling occupational accidents.

A qualitative content analysis was conducted to develop an in-depth understanding of major organizational factors affecting occupational accidents. According to qualitative research procedure, sampling in this study was purposive, and the sample size was based on data. Purposeful sampling is widely used in qualitative researches for the identification and selection of information-rich cases related to the phenomenon of interest (17, 18).

Purposive sampling was used to select 17 participants with abundant information. Four of the experts selected were female. The participants were selected from various positions in university and petrochemical complexes in the Iranian cities of Tehran, Esfahan and Mahshahr. Further characteristics of the participants are presented in Table 1.

**Table 1:** Participants characteristics (n=17)

**Participant Number**	**Age (yr)**	**Gender**	**Years of Experience**	**Ward**	**No. Of Interviews**	**City**
1	40	Male	10	University professor	1	Tehran
2	63	Male	29	University professor	1	Tehran
3	57	Male	25	University professor	2	Tehran
4	65	Male	31	HSE supervisor	1	Tehran
5	53	Male	15	University professor	1	Esfahan
6	41	Female	12	University professor	1	Tehran
7	44	Male	10	HSE supervisor	2	Mahshahr
8	55	Female	12	University professor	2	Tehran
9	39	Female	9	HSE supervisor	1	Tehran
10	31	Male	7	University professor	1	Esfahan
11	39	Male	12	HSE supervisor	1	Esfahan
12	41	Male	10	HSE supervisor	1	Mahshahr
13	45	Male	12	University professor	1	Tehran
14	37	Female	7	HSE supervisor	1	Tehran
15	44	Male	11	Head of Unit	1	Mahshahr
16	40	Male	9	Head of Unit	1	Mahshahr
17	45	Male	12	Head of Unit	1	Mahshahr

The data were collected using semi-structured interviews. The participants had at least five years of experience. A researcher who was familiar with the principles of qualitative approach and had practical experience in qualitative investigation carried out interviews.

The study began on the subject with some general questions. In addition, consultants and expert’s opinions were used in the design of questions. Some of the questions were pre-designed according to the purpose of the study. Moreover, some deep and exploratory questions (e.g. explain why and how) had been set based on the type of answers to understand the depth of perception and views of the participants. The questions were about the factors in occupational accidents. For example, of the Scientific Board members were asked: “*What actions can be done to prevent accidents in an organization?*” On the other hand, the safety and health supervisors were asked: “*What strategies a manager can use in an organization to control occupational accidents*?”

Several encouraging questions were also made for the participants to express their experiences in this field.

Each interview lasted between 60 and 80 min and was performed in the participant’s office in academia and industry. The second interview with three participants was conducted for verifying and clarifying the elementary interpretations of the data and emerging the research findings. To ensure the accuracy of the data, the interviews were continued until data saturation. A collection of data was ended when no new information could be collected and the data became repetitive (19).

The interviews were recorded and transcribed verbatim with the participants’ permit and then coded according to the content analysis method.

Code management was done with the help of MAXQDA-10 that is professional software for qualitative and mixed methods’ data analysis (20). The various codes were compared and classified, and the themes were created upon similar and suitable categories.

During the study, some procedures were used to certify the data trustworthiness. The participants were ensured for keeping the names confidential and authorization was taken from the industrial authorities. For credibility and conformability, a member checked the raw data (in this case, codes extracted for accuracy of the meanings’ interpretation were returned back to some participation and verified by them).

## Results

Eleven organizational factors’ sub-themes were identified: management commitment, management participation, employee involvement, communication, blame culture, education and training, job satisfaction, interpersonal relationship, supervision, continuous improvement, and reward system (Table 2). The participants considered these organizational factors effective in occupational accidents.

**Table 2:** Identified organizational factors: themes, sub-categories, and codes

**Theme**	**Sub-themes**	**Codes**
	Management commitment	Provide financial, personnel and time resources Management support Set up training courses
	Management participation	Management must be involved in safety and health programs. Management should participate in safety meetings
	Employee involvement	Managers should actively think about what the employees suggest Near miss report Use of personal protective equipment
	Safety communication	Regular meetings, and suggestion box Workers should share their views with the management Top-down and down-top form
	Blame culture	Create a culture of prevention of errors Report without fear of the consequences Error acceptability
Organizational factors	Education and training	Lack of training is one of causes of high rates of accidents. Safety training enhances the knowledge and motivation Safety training improves the safety climate Evaluating the effectiveness of courses is important.
	Job satisfaction	The motivation to do the work working conditions, salary, opportunities for career advancement
	Interpersonal relationship	Supervisors are in direct contact with employees. Employees feel that their opinions are important for the supervisor.
	Safety supervision	Contractors should be under regular supervision
	Continuous improvement	Current situation is not completely satisfying. Have a long-term plan to improve the safety situation
	Reward system	Financial and non-financial gifts Create motivation

### Management commitment and participation

According to the participants, management commitment is very effective in reducing occupational accidents. Participant experience showed that management commitment to safety creates motivation throughout the organization. Most of the participants expressed that this commitment can show itself through providing financial, personnel and time resources, as well as management support and safety measures such as trying to set up training courses. One expert believes that “*only financial fund management is not sufficient in the promotion of safety and management must be involved in the safety and health programs*”. A HSE supervisor with 12 yr of experience said: “*Management must show to the employees that the importance of safety and health is more than production, safety first must be truly implemented in the workplace. I think to achieve this manager must give importance to the considerations of safety and health in their decision making*”. Other participant mentioned that “*I know are fiery manager who, when the work starts every morning, wears his uniforms and hats and safety shoes, goes around the entire site; makes greeting with all workers and then goes to his working office*”.

### Employee Involvement

One of the factors that the experts pointed out is employee involvement. One participant stated: “*In daily work conversations, health and safety issues must be considered, and the managers should actively think about what the employees suggest*.” Another participant said: “*Perhaps the best advantages of worker involvement in safety issues are reported near miss. If the employees feel that their talk is important for safety, they are encouraged to become involved in safety issues*”. One of the participants said: “*Workers use knowledge of their surroundings to understand what things are putting people in hazardous situations. Workers need control to make appropriate changes to the system. Finally, workers’ ideas and proposals need support from the management to enable them to feel they can truly make a difference in the lives of their coworkers*”.

### Safety communication

One of the safety and health managers believed that “*this relationship should be both in top-down and down-top form*”. Workers should be able to share their views with the management. Means in this item can be important, and the best is holding regular meetings with the workers’ representatives with the management or suggestion box. When managers and workers in both formal and informal settings regularly discuss expectations and subjects, all team members have a better understanding of the condition of the working relationship. Regarding the communication, one of the participants stated: “*Management should show feedback to the demands of personnel. If employees know that expressing their demands is useless, then there would be no desire in them to report issues. While one worker may be responding to incoming complaints per company policy, another employee may really have a solution to the subjects causing the complaints, such as a product modification*”.

### Blame culture

In relation to blaming culture, the organization should create a culture of errors’ prevention, and provide a suitable environment for employees to report errors without fear of the consequences. One of the participants said: “*To achieve this goal, organizations need to organize and manage errors creation risk management system based on the culture of error of acceptability*”. Another participant said: “*In an organization where people are afraid of telling the truth, then you cannot expect they report near miss and accidents. And the risk sources continue to exist in such organization and cause to accidents occasionally during the time”*.

Other participant mentioned, “*Blaming the worker is a traditional approach; this leads the worker to lose his/her motivation, become disillusioned, and may commit retaliatory actions. When an accident occurs, one must consider the reasons of accident. Worker blame results in non-reporting and under-reporting of problems*”.

### Education and training

Another identified organizational factor contributing to occupational accidents is personnel education and training. All of the participants remembered the lack of education and training as a critical organizational factor. One expert said: “*lack of training or inadequate training of workers is one of causes of high rates of accidents*”.

Safety training enhances the knowledge and motivation, and thus improves the safety climate. Therefore, effective safety training through determining educational safety needs, using optimal training methods and evaluating the effectiveness of courses has an important role in strengthening safety culture, reducing accidents and consequently controlling injuries and damages. One participant said: “*Training is effective when the system has no hardware problem and problems that are in your workplace be unsafe working behavior”. He also added, “Education would cause to a change of attitude and behavior*”.

### Job satisfaction

Job satisfaction was mentioned as one of the main causes of occupational accidents. Job dissatisfaction is one of the factors in the work leave. Job satisfaction factor affects the performance and safety behavior of the employees. Experts of industrial management said, “*Job satisfaction is a multidimensional concept, and is associated with many factors. In other words, job satisfaction will be achieved with the implementation of a set of factors; these factors include the nature and working conditions, salary, opportunities for career advancement, quality supervision, and relationships with colleagues and others*”. One participant said: “*Workers, who have more job satisfaction, more quickly learn new tasks associated with the job, and are faced with lower job accidents. The salary for almost all job groups is considered one of the most important working conditions. In cases, it is seen that job safety has been affected because of low salaries and lack of incentives*”.

### Interpersonal relationship

This item refers to the relationships between workers and supervisors. Supervisors and workers can discuss any issue openly in the workplace. One participant stated: “*Supervisors are in direct contact with employees, therefore, they can have an impact on safety-related behaviors such as participation in safety issues. Supervisors should behave in a manner that the employees are involved in safety activities, and feel their opinions are important for the supervisor*”. In addition, some participants said that to achieve this objective appropriate educational program should be implemented for supervisors. One participant said: “*Supervisors can improve the organization’s climate by bringing the employees closer together, thereby reducing friction and misunderstandings*”. Another participant mentioned “*Supervisors are bridge between managers and employees, and their behavior must be such that employee trust and do not have fear to express their problems*”.

### Safety supervision

Work environment safety supervision is one of the key factors in the prevention of occupational accidents. One head of unit in a petrochemical complex stated: “*Naturally, if there is no supervision in any activity, you cannot expect progress at that work, and safety is not an exception*. *For effective supervision of workplace safety and health, supervisors need to be trained*”. Another participant said: “*Especially in the case of contractors, there should be regular supervision plan, as lack of oversight of contractors can ignore safety and health requirements, especially in where there is a need to add to the work speed*”. One of the participants said: “*Supervision is not telling somebody what to do and how to do it. Safety supervision is a legal responsibility under occupational safety and health laws. Supervision is to make sure the employees are following safety instructions and working safely*”.

### Continuous improvement

Another organizational factor affecting occupational accidents is continuous improvement. This means that the current situation is not completely satisfied, and we must always seek to improve. Continuous improvement should include all individuals, including managers, employees, and workers. One of the safety managers said: “*Organization should be having a long-term plan to improve the safety situation, and the goal is reached when corrective actions are planned and implemented*”. One participant said: “*I think an occupational health and safety management system is required in order to eliminate or reduce the risks to personnel and other interested parties exposed to health and safety hazards arising from the operations of the organization. By the safety management system achieve to continuous improvement can by performing corrective measures*”.

### Reward system

This system might create motivation to employees for safe behaviors. If this action is not handled properly, it can cause unsafe behaviors among people. One of the participation said: “*There are two basic types of rewards, financial and non-financial, and both can be applied positively to amplify performance behaviors of the workers. Financial rewards can be in terms of incentives, salary/pay, promotions, bonuses, job security, etc. Non-financial rewards are psychological rewards like appreciation, positive and caring attitude from employer, meeting the new challenges, and job rotation after attaining the goal*”.

## Discussion

This study aimed to investigate the organizational factors affecting occupational accidents. Experiences and perceptions of the participants have been placed in eleven sub-categories: management commitment, management participation, employee involvement, safety communication, blame culture, education and training, job satisfaction, interpersonal relationship, safety supervision, continuous improvement, and reward systems.

Management’s commitment to safety is a major factor of safety climate, which is a subset of organizational factors and implies the extent to which top management shows supportive and positive attitude toward safety (14, 21). Management commitment was indicated to safety is the main factor affecting the success of the safety program. This commitment will be shown through job training programs, management involvement in safety committees, attention to safety in the job design, and review the pace of work (22). As the participations pointed out, management commitment is not limited only to adequate financing; rather the management should have a tangible presence in other safety-related activities too.

The experts stated that Workers’ involvement has many advantages. These findings are consistent with the reports of several studies. For example, the policy of workers’ participation was pointed out, including employees’ involvement in decisions about their work, makes sense to encourage them to offer suggestions, which in turn can remove much inefficiency related to poor job design (23). Employees’ participation requires an open environment where people can present their own ideas without blame (24). Good safety culture “is where the mental attitude of employees and management is in such a way that when understanding a risk to health and safety, quickly report to designated individuals. “An organization must necessarily encourage consciousness and investigation of its members, rather than punishing the messenger” (25).

The biggest disadvantage was not reporting accidents and near misses. This culture makes employees avoid from telling the truth. Blame culture refers to the proneness of management to punish workers when they do make fault. These findings are consistent with the report that suggested blame culture may disappoint workers from reporting workplace safety problems, resulting in a negative impact on the workers’ safety performance (1). Workers to avoid management blame or punish may selectively decide what news to report, share good news and conceal problems. In the space of no blame in an organization, employees created with high performance in a powerful organization, and they increase the efficiency and productivity (15).

The most important factor creating occupational accidents as mentioned by the participants was referred to the training. Priority for education and training is part of a safety culture (26). Education is a modifier ongoing process. Safety must be an integral part of a training program. Education must tighten safety awareness and intelligence comprehension of risks. The role of education in safety climate was examined and concluded that safety training as a powerful mechanism has a positive effect on safety climate as a subset of organizational culture (27); this point was mentioned in the statements of several participants.

Another organizational factor that its relationship to safety performance has been shown in different studies is job satisfaction (28, 29). Job satisfaction was associated with incident rate, and the higher JDI satisfaction scale caused lower incident rate (30). Besides, in our study, the participants pointed to the relationship between job satisfaction and occupational accidents. Employee-supervisor relation affects the safety-related behaviors of employees such as compliance and participation. Employees work more safely when they were involved in the processes of decision-making or receiving immediate feedback about their work (31). An improvement in employee safety behaviors like wearing protective equipment when the supervisor spoke about safety with them in a more open manner (32). This study has provided further evidence on the importance of supervision in the management of safety. Four items of supervisor safety management were discovered to be critical. Those were valuing subordinates; visiting the work site regularly; a participatory method of management and efficient safety communication. The subordinates of supervisors who show these behaviors most frequently are less likely to be involved in an accident (33). The effect of reward system has been mentioned on safety awareness and safety behavior (14, 34). A reward system can be performed by utilizing extrinsic and intrinsic rewards. Extrinsic rewards are salary, bonus, and fringe benefit while intrinsic rewards involved praise, encouragement, and empowerment. By utilizing positive amplification in these factors, favorable positive behaviors are encouraged, and negative behaviors are omitted (35). The present research participants in connection with the reward system also believed that this system might create motivation to employees for safe behaviors.

## Conclusion

There are many organizational factors involved in occupational accidents. The most important items among these can be noted as follows: management commitment, management participation, blame culture, education and training, job satisfaction, safety supervision, and reward system. Organizational factors influence the safety-related behaviors and safety performance. Accordingly, the attention of the industrial authorities for the required action should seek to reduce the occupational accidents. A qualitative study should be done for investigating the environmental and individual factors in this regard. Moreover, it is recommended to conduct a study to identify practical solutions to reduce the workers’ occupational accidents. The low sample number in this study may limit the generalization of the survey results. Of course, this is considered as the inherent limitation of qualitative studies.

## Ethical considerations

Ethical issues (Including plagiarism, informed consent, misconduct, data fabrication and/or falsification, double publication and/or submission, redundancy, etc.) have been completely observed by the authors.
